# Systematic review on quality control for drug management programs: Is quality reported in the literature?

**DOI:** 10.1186/1472-6963-9-38

**Published:** 2009-02-25

**Authors:** Anke-Peggy Holtorf, Carrie McAdam-Marx, David Schaaf, Benjamin Eng, Gary Oderda

**Affiliations:** 1 Department of Pharmacotherapy, The University of Utah, Millcreek Outcomes Group, Salt Lake City, UT, USA; 2 Primary Care US Medical Affairs, Pfizer Inc., New York, NY, USA; 3 Specialty US Medical Affairs, Pfizer Inc., New York, NY, USA

## Abstract

**Background:**

Maintaining quality of care while managing limited healthcare resources is an ongoing challenge in healthcare. The objective of this study was to evaluate how the impact of drug management programs is reported in the literature and to identify potentially existing quality standards.

**Methods:**

This analysis relates to the published research on the impact of drug management on economic, clinical, or humanistic outcomes in managed care, indemnity insurance, VA, or Medicaid in the USA published between 1996 and 2007. Included articles were systematically analyzed for study objective, study endpoints, and drug management type. They were further categorized by drug management tool, primary objective, and study endpoints.

**Results:**

None of the 76 included publications assessed the overall quality of drug management tools. The impact of 9 different drug management tools used alone or in combination was studied in pharmacy claims, medical claims, electronic medical records or survey data from either patient, plan or provider perspective using an average of 2.1 of 11 possible endpoints. A total of 68% of the studies reported the impact on plan focused endpoints, while the clinical, the patient or the provider perspective were studied to a much lower degree (45%, 42% and 12% of the studies). Health outcomes were only accounted for in 9.2% of the studies.

**Conclusion:**

Comprehensive assessment of quality considering plan, patient and clinical outcomes is not yet applied. There is no defined quality standard. Benchmarks including health outcomes should be determined and used to improve the overall clinical and economic effectiveness of drug management programs.

## Background

An ongoing challenge in the United States (USA) healthcare system that all stakeholders face is maintaining quality of care while managing limited healthcare resources. Meanwhile, accreditation and professional organizations facilitate provider and payer quality improvement efforts by offering quality measurement and tracking initiatives.

Some of the most widely recognized healthcare quality measurement programs include accreditation and quality reporting of the National Committee for Quality Assurance (NCQA) which is based on the Healthcare Effectiveness Data and Information Set (HEDIS) and the Joint Commission on Accreditation of Healthcare Organizations (JCAHO) Performance Measurement initiatives. [1]

While measures of adherence to prescribing recommendations for specific diseases or conditions are incorporated into HEDIS and JCAHO standards, they are not specifically designed to measure the quality of drug management programs. Drug management programs and tools are generally implemented by healthcare payers and health systems to manage and control the use of prescription drugs, with the ultimate goal to help ensure effectiveness. This allows available resources to be used to deliver desired health outcomes effectively. Thus, it is important to monitor the impact of these programs to ensure that drug management programs do not unintentionally lead to negative patient outcomes as poor outcomes may in turn increase overall medical costs.

Several efforts are underway to establish recognized quality measures for pharmaceutical care in various environments, including the payer/managed care setting. The Academy of Managed Care Pharmacy (AMCP) Formulary Submission Format is one initiative specifically focused on drug management programs. The AMCP Format is a guideline used by pharmaceutical manufactures for compiling clinical and economic data used by payers in making evidence-based, value driven formulary decisions. [2]

Pharmaceutical components are included as part of a more comprehensive quality program. One example is the National Quality Forum (NQF), a multi-stakeholder organization that is building consensus for measuring and reporting healthcare quality across a variety of settings, including therapeutic drug management. [3] In addition, the National Business Coalition on Health (NBCH) has created the eValue8 program which is used by business coalitions to compare health plans across a variety of performance measures, including pharmacy programs. [4] Finally, URAC, which includes a wide range of stakeholders (employers, consumers, pharmacy consultants, health plans, independent retail pharmacy, pharmacy benefits management (PBM) organizations, pharmacy professional organizations, labor, and large public purchasing groups) has also devised a Pharmacy Benefit Management Accreditation Format, which was made available in 2007 and aims to be a key benchmark for quality of care. [5]

These quality efforts are a start to measuring and evaluating quality for prescription drug management programs. However, the stakeholders have not yet agreed on recognized standards and benchmarks for determining how the quality of drug management programs should be defined and measured. Without such standards and transparent performance reporting for drug management programs, it is difficult for payers, providers, policymakers and patients to establish or evaluate quality of drug management programs.

Numerous reviews have been conducted to synthesize the findings of drug management program research conducted in the USA, including an assessment of economic findings by Hadley et al. [6] and a recent review of the association between cost sharing, medication utilization, and outcomes found in the literature by Goldman et al. [7] However, it appears that no published studies have reviewed the methodological approaches taken in the US to assess how program impact is measured across a range of drug management tools to identify if evaluation trends exist. Nor is there a review of how plans and researchers use this data to assess program outcomes and quality.

Thus, the aim of this analysis was to evaluate how the impact of drug management programs is quantified as reported in the literature over the last 12 years and to identify whether predefined thresholds for quality are utilized in these analyses. Such information will help identify trends that can build towards a consensus for assessing quality in payer-based drug management programs in the US.

## Methods

A librarian assisted search of the literature was conducted of articles published in English from 1996 to 2007. The time period of 12 years has been chosen to be able to identify measurement processes related to drug management tools currently utilized and to see potential changes in the publications over time. The databases searched include PubMed, International Pharmaceutical Abstracts (IPA), CINAHL, and Business Source Premier. In addition, meeting abstracts from the International Society of Pharmacoeconomic and Outcomes Research (ISPOR) and Academy of Managed Care Pharmacy (AMCP) were reviewed. Search terms, which are listed in table 1, were used alone and in combination to identify potential articles. In addition, the reference lists from key studies and related review articles were reviewed to identify studies not picked up in the automated search process.

**Table 1:** Search terms for literature retrieval

Search Terms	
Primary MeSH Headings	

Insurance, Pharmaceutical services	Drugs, non-prescription

Managed Care Programs	Drugs, Generics

Pharmaceutical Preparations	Prescriptions, Drug

Additional MeSH Headings	

Formularies	Total Quality Management

Pharmacy and Therapeutics Committee	Outcome Assessment (Health Care)

Drug Utilization Review	Cost Control

Deductibles and Coinsurance	Cost Sharing

Reimbursement, Incentive	Community Pharmacy Services

Additional MeSH Sub Headings	

Classification	Standards

Economics	Statistics and numerical data

Methods	Trends

Additional Free Text Search Terms	

Drug Management Programs	Step-edit

Drug Benefit/Pharmacy Benefit	Therapeutic Substitution

Formularies	Medication Therapy Management

Formulary Process	Drug Therapy Management

*tier Copay (e.g. three-tier copay, four-tier copay)	Pharmacy Cognitive Services

Formulary Restrictions	Quality

Generic Drug Program/Mandatory Generic Prescribing Program	Outcomes

Prior Authorization (Prescription Drug)	Costs/Resources

Quantity Level Limits	Patient Reported Outcomes

Abstracts and articles generated from original research studies, meta analyses, and other systematic reviews were reviewed by a pharmacist and pharmacoeconomics outcomes researcher for inclusion based on the following criteria: (1) Studies must have been conducted in a managed care, indemnity insurance, VA, or Medicaid setting in the US; (2) Studies must have evaluated the impact of at least one drug management tool including overall drug formulary/preferred drug list, drug use review, copayment/cost-sharing, prior authorization, coverage restrictions including specific formulary restrictions, step edits, therapeutic interchange, generic programs, and/or combinations of the above; (3) Articles must have reported either processes, economic, clinical, or humanistic outcomes. If there was ambiguity among the two reviewers, the study was included or excluded based on a discussion and subsequent consensus. The results of the analysis were documented in an Access database, which had been specifically developed for the comparative analysis and evaluation in this study. This database was used by both reviewers for review and quality control.

All articles were systematically analyzed for the main study objective, study endpoint parameters, the drug management type, and other criteria. The review of the studies revealed that the primary mechanisms of the drug program management tools fell into three broad categories: (1) shifting 'financial responsibility to patient' (including overall formulary programs, preferred drug lists, and patient cost sharing measures), (2) 'limit or control access' (including specific formulary restrictions, prior authorization, step edits, and quantity level limits), and the (3) 'stimulation of switch to preferred agents' (including therapeutic interchange, generic drug incentives, and over-the-counter drug incentives). Similarly, the study endpoints represented four different perspectives: (1) 'plan focus' (including plan cost and drug utilization), (2) 'patient focus' (including patient out-of-pocket cost and patient satisfaction), (3) 'clinical focus'(including compliance and persistence, likelihood of receiving treatment, clinical outcome, and medical resource utilization), and (4)'provider-focus' (including adherence to treatment guidelines, prescription switching, and workload for provider). If a study evaluated two related drug program tools, the study was categorized on the basis of each program type. A small number of studies (n = 7; 9%) evaluated more than two distinct programs (multiple drug management type). Studies that evaluated multiple endpoints were included in each study endpoint category.

## Results

The literature review identified 1099 potential articles. Titles and abstracts of these articles were reviewed to assess if the studies met the established inclusion criteria. From this initial review, 193 articles were pulled for in-depth review; 76 articles met all inclusion criteria and were included in the assessment. These articles are listed in Additional file 1[8-83]. An increase in the number of studies can be seen throughout the time of our analysis; there were three studies published before 1999, four in 1999–2000, thirteen in 2001–2002, twenty-four in 2003–2004, and thirty-two in 2005–2007.

### Primary drug management tools evaluated

None of the studies assessed quality related measures for an overall drug management program. Rather, studies examined the impact of 9 different drug management tools alone or in combination.

**Figure 1:**
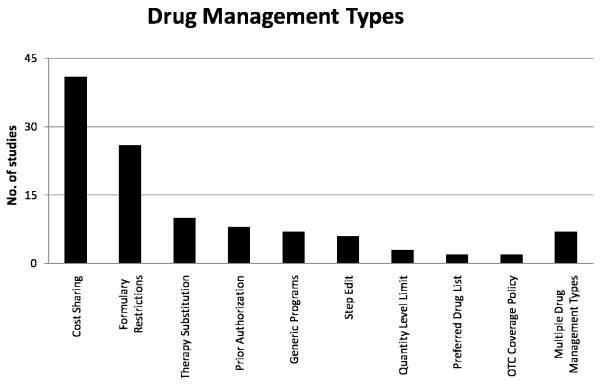
**Drug Management Type**. Number of studies analyzing the impact of each drug management tool (total = 76).

A majority of the studies (43 studies; 57%) evaluated drug management tools that shift financial responsibility for non-preferred agents to patients through full or partial cost sharing. Programs that drive use of preferred agents by stimulating a switch in prescribing or utilization to preferred agents (8 studies; 14%) were the least common of the three program types evaluated. The specific drug management tool most frequently evaluated was cost sharing (41 studies; 54%). These studies assessed the impact of multiple tier formularies or coinsurance programs (Figure 1). The least evaluated drug management program tools were OTC coverage policies and overall preferred drug list/formulary evaluations (each with 2 studies; 3%).

### Study endpoints

**Figure 2:**
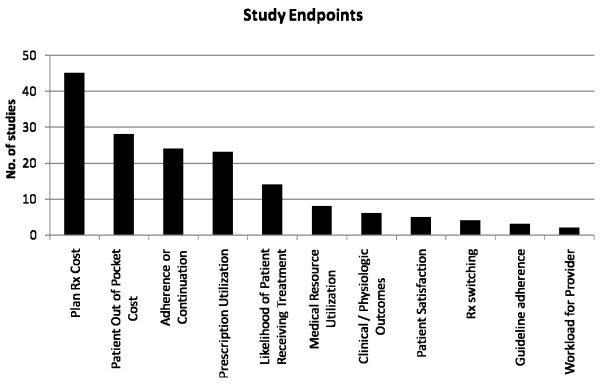
**Study Endpoints**. Number of studies analyzing specific endpoints.

Rx cost was the most frequently used endpoint (in 45 of 76 studies) followed by patient out-of-pocket cost (28 of 76), and medication adherence (24 of 76) (figure 2). Only 7 of the 76 studies, thus less than 10 percent, account for a clinical or physiological disease related endpoint. An average of 2.1 (+/- 1.1) endpoints were evaluated per study. A majority of 49 studies (64%) looked at one or two endpoints (Table 2).

**Table 2:** Endpoint breadth used for different drug management tool categories

Number of Endpoints	1	2	3	4	5	Number of Studies	Average No. of Endpoints	St. Dev
Drug Management Tool Category								
Financial responsibility to patient	16	8	13	4	2	43	2.3	1.2
Limit access to non-preferred agents	17	12	8	2	1	40	2.0	1.0
Stimulate switch to preferred agents	6	2	0	0	0	8	1.3	0.5

All Drug Management Programs	29	20	18	6	3		2.1	1.1

Analysis by number of endpoints included in the studies in each drug management tool category (n = 76)

The predominant study endpoint category utilized in evaluating the impact of drug management program tools was plan focused (53/76 studies, 68%; Table 3). Within this category, a plan drug cost endpoint was reported in 47/76 studies; 62%) followed by drug utilization (23/76 studies; 30%).

**Table 3:** Relation between Drug Management Tool Objective and Study Endpoints

Drug Management Program Categories		**N**	**Plan focus**	**Patient Focus**	**Clinical Focus**	**Provider Focus**
	**n**	76	53	33	35	9
	**%**		70%	43%	46%	5%

**Financial responsibility to patient**	**n**	43	30	27	20	2
	**%**		70%	63%	47%	5%
**Limit access to non-preferred agents**	**n**	40	28	13	15	6
	**%**		70%	33%	38%	15%
**Stimulate switch to preferred agents**	**n**	8	6	3	0	1
	**%**		75%	38%	0%	13%

Number of Studies by Drug Management Tool Category and Study Endpoint Type: The percentages relate to the number of studies in that drug management program category. Studies with several endpoints can be in multiple endpoint categories, studies comparing different drug management programs can be in multiple drug management program categories.

Clinical focused endpoints was the second highest endpoint category evaluated. A total of 35 of the 76 reviewed studies assessed clinical endpoints (45%) including compliance and persistence (24 studies), likelihood of receiving treatment (14 studies), and clinical outcomes (7 studies). Patient-focused endpoints was the third most frequent endpoint category evaluated (33 of 76 studies; 42%). This category included patient out-of-pocket costs (28 studies) and patient satisfaction (5 studies). Provider-focused endpoints were evaluated in 8 studies (11%) which included prescription switching (4 studies), guideline adherence (3 studies), and provider workload (2 studies).

Evaluations of drug management programs that shifted financial responsibility to the patient (43 studies) predominately measured plan focused outcomes (30/43 studies; 70%) followed by patient-focused outcomes (27/43 studies; 63%). Less than half evaluated a clinical focused outcome (20/43 studies; 47%). A majority of assessments of tools that limit or control access through benefit restrictions (40 studies) included plan focused outcomes (28/40 studies; 70%). Less than half evaluated a patient-focused outcome (16/40 studies; 40%) or a clinical focused outcome (15 of 40 studies; 38%). A similar proportion of the 8 studies of drug management program tools that stimulated a switch in prescribing or utilization to preferred agents evaluated plan focused outcomes (6 studies; 75%) and patient-focused outcomes (3 studies; 38%), however none of these studies evaluated clinical outcomes.

Across studies of drug management program tools, there are examples where differences in conclusions may have been caused by a lack of consensus on defining outcomes. Two examples are discussed below.

A total of 14 of the 41 studies (34%) of drug management program tools that involved cost sharing with the patient such as tiered formularies and coinsurance, evaluated treatment adherence as a study endpoint. Adherence was measured by whether patients continued to take their medication (persistence; 5 studies), by whether patients took their medication as prescribed (compliance; 6 studies) or both (2 studies).

When persistence was evaluated, higher cost sharing had no effect (5 studies)[15,23,56,59,79] or mixed effects (1 study). [63], with only one study finding that higher cost sharing negatively impacted medication continuation. [39] When compliance was evaluated the opposite was identified. Seven studies found that compliance decreased with higher cost sharing [15,29,33,47,54,75,78], and two studies found that compliance increased with lower cost sharing. [13,50] The second example relates to studies that evaluated step edit programs by the impact on plan costs. [58,66,80,82] The studies differed by whether administrative costs were considered in addition to drug costs. When only drug costs were considered, all four studies concluded that step edits drove cost savings for the plan. However, one study also included administrative costs, and in this case the authors concluded that step edits did not deliver a cost savings to the health plan. [66]

### Benchmarks

The published evaluations of drug management programs generally did not utilize pre-defined goals for assessing the quality of the program evaluated. In one exception, Monane et al studied the impact of a pharmacist intervention program in which physicians were contacted regarding possible medication related problems in the elderly. [55] This study identified a prescribing change benchmark of 2% as the minimum target, which reflects the average rate of prescribing change in physician practice overall.

## Discussion

This analysis evaluated studies that quantified the impact of drug management program tools on health plans and patients. The purpose of this study was to identify how management programs are evaluated and whether predefined thresholds for quality are utilized.

Overall, studies of drug management programs are relatively limited in scope. While 11 endpoints were used across the literature most studies focused on one or two endpoints (64% of the studies). The predominant perspective was that of the plan, with 53 (70%) of the studies analyzing a plan focused outcome. This trend reflects the fact that most drug management tool evaluations are conducted and published by health plans, which would clearly have a vested interest in measuring how drug management program tools impact the plan financially.

Drug management programs also impact patients financially or by limiting the range of drugs that they can access with their pharmacy benefit. However, just less than half of the identified studies included a patient-focused outcome relating to out of pocket costs, or patient satisfaction (n = 33; 43%). Similarly, less than half of the identified studies included clinical endpoints (n = 35; 46%). Thus, most drug management tool evaluations are not yet measuring the full impact to plans and patients in terms of total healthcare costs and clinical outcomes. [84] Austvoll-Dahlgren et al. performed an international systematic review of the effects of coverage caps and co-payments on rational drug use and concluded that introducing or increasing co-payments reduced drug use and saved plan drug expenditures. However, the authors point out a lack of discussion in these articles on the intensity of the caps and copayments, and similar to this review, that there was little assessment of their impact on health outcomes or continuation of treatment.

In our analysis, studies of drug management programs that shift costs to patients tended to be more comprehensive. However, just over half of these studies included a patient-focused or clinical endpoint in addition to a plan-focused endpoint. A move towards more comprehensive quality analyses is not yet seen with evaluations of other drug management tool categories. Notably, only a third of program evaluations of drug management tools that limit or control access through benefit restrictions included patient-focused or clinical outcomes. Program evaluations of tools that enforce or encourage switching to preferred agents exhibited the highest proportion of studies that included a plan-focused outcome. While the proportion that included a patient-focused outcome was similar to the other drug management tool categories, none of these studies included a clinical outcome.

Provider-focused endpoints were the least evaluated, with the greatest proportion of evaluations that included a provider-focused endpoint were those of cost shifting drug management program tools. This is an interesting observation as benefit design limits may mandate adherence to prescribing of preferred agents. Such programs could also result in no medication being prescribed, thus provider behavior endpoints measuring adherence with treatment guidelines is warranted. However, only 6 of the 40 studies on limiting access to non-preferred drugs evaluated prescriber behavior, and of those only 2 assessed adherence with treatment guidelines.

It was interesting that evaluations of drug management programs that shift costs to patients were more likely to evaluate patient-focused and clinical endpoints than evaluations of programs that limit or control access through benefit restrictions or by stimulating a switch in prescribing or utilization to preferred agents. This trend may be related to concerns that shifting costs to patients may have the unintended consequence of causing patients to not take medications as prescribed and therefore may result in negative clinical or humanistic outcomes. Hence, this group of studies reflects the point of view of a different set of stakeholders. The considerations of patient and clinical endpoints in addition to plan endpoints may reflect an evolution of drug management program evaluations. However, only less than 10% of the studies included a disease related clinical or physiological outcome. Considering, that the improvement of the clinical or physiological level should be at the core of healthcare, there appears to be a large gap in studying the impact of drug management programs. A study by Hodgkin et al. on the impact of three tiered formularies on antidepressant utilization and expenditures notes that the health economic consequences and quality impact of multi-tiered formularies may depend on the therapeutic area. Non-differential cost shifting to patients may limit individual therapy adaptations. [85] These authors, therefore also suggest tracking the effects of three-tier programs on patient adherence, quality of care, and clinical and economic outcomes.

In addition to forming a consensus on which endpoints are most appropriate to evaluate for specific drug management tools, it is also important to obtain and maintain agreement on how specific endpoints are evaluated. This study defined endpoints in fairly specific terms with the purpose of categorization. Even with such specific endpoint definitions two examples of endpoint disparity were evident: in (1) medication adherence (medication compliance and persistence) as an endpoint in cost shifting drug management programs and (2) plan costs in evaluations of step edit programs. In these cases, differences in measurement approaches led to different conclusions. Thus, to ensure that plans, payers, and patients can compare and contrast the quality of different or similar programs there a need to reach consensus on what is being measured as well as how the endpoints are measured.

In addition, a conspicuously absent element of drug management program studies was the lack of predefined targets or recognized quality benchmarks. While studies were qualified in terms of endpoint measures, such as 'did or did not reduce costs', 'did or did not impact medication compliance', these evaluations tended to describe outcomes without specifying the ideal target a priori and what would constitute an acceptable range of outcomes. Only one study identified a prescribing change benchmark/goal which was based on an estimate of the average percentage of prescribing that differs in a prescriber from one year to the next. However, this article failed to justify the selected 2% benchmark versus a higher rate that may have been more indicative of prescribing changes in the presence of known clinical risks. Thus, while any article utilizing a benchmark should be commended, it is equally important that the benchmark be justified for the given setting and program objective.

Overall, this analysis has identified that, currently, there is a lack of consistency in how drug management program tools are evaluated. Furthermore, drug management tool evaluations tend to fall short of comprehensives assessments and they do not identify and apply comparative benchmarks. Drug management program tools often considered costs to the health plan or patient costs or satisfaction. However, a minority of the evaluations incorporated an overall assessment of clinical outcomes or medical utilization to ensure that programs support the efficient and effective use of health plan resources. In other words, few drug management program tool assessments measure value as defined by the relation between quality of care and efficiency of care. [86] Our observation is confirmed by the findings of Lu et al who reviewed the impact of interventions targeting drug use in the US managed care setting. [87] Despite evidence for the effectiveness of several strategies in changing drug use in the managed care environment, the most studies did not provide evidence for the cost-effectiveness of the interventions. Similarly, Ovsag et al. raise the issue of compromising long term quality of care and overall economic impact for the advantage of expected short-term cost savings in the context of preferred drug lists in the Medicaid environment. [88]

In April 2002, the Academy of Managed Care Pharmacy (AMCP) introduced the Pharmacy's Framework for Drug Therapy Management in the 21st Century. In this, the working group of more than 100 drug therapy specialists agreed on the following measurements for improving healthcare quality: (a) a better patient outcome at the same cost, (b) the same patient outcome at lower cost, (c) a better patient outcome at lower cost, or (d) a significantly better patient outcome at moderately higher cost. [89] Consequently, any form of quality assurance has to integrate cost and patient outcome monitoring and will not be complete without integrating multiple perspectives.

While this analysis begins to identify an information void regarding the quality assessment of management programs, the analysis has several limitations that warrant mention. First, the published literature is likely to lag real-world activities related to the quality assessment of drug management programs. Thus, the published literature may not yet reflect the cutting edge or detect emerging trends in the quality assessment of drug management programs. Continued publication of these evaluations should be highly encouraged to share best practices and facilitate consensus on quality assessment approaches for drug management program tools.

Additional limitations arise from the study design, including the somewhat subjective categorization of drug management tool types and study endpoints. Different categorization approaches may have modified specific study findings and conclusions. However, it is unlikely that different means to describing program evaluations would have changed the primary conclusions regarding the current lack of consensus on program tool evaluations and lack of benchmarks for determining the quality of drug management program tools as is also confirmed by other upcoming publications in this context. [84,87-89]

In this review, we have analyzed the methods used by researchers to study the impact of drug formulary management tools and whether there is any indication that quality assessment standards have emerged. To illustrate the risk of inconsistency in the quality assessment of drug management program tools we selected two examples of how different methods of endpoint evaluation clearly impacted conclusions. While the methodological question is the focus of this paper, it will also be important to understand the actual impact of the diverse drug formulary management programs. Such a review should be much more in depth than the isolated examples used here and thus, it is planned as an additional extended step of this study.

## Conclusion

In summary, the literature evaluating the impact of drug management programs has revealed that trends in how to measure the impact and quality of drug management programs are starting to emerge. However, consensus is not yet building uniformly across types of drug management programs, and a comprehensive assessment of quality considering plan, patient and clinical focused outcomes is not yet applied. Furthermore, there is no evidence in the literature that researchers and health plans have identified and recognized benchmarks or goals that programs should be striving to achieve to ensure that efforts to manage drug benefit resources are not compromising patient outcomes. More than 90% of studies do not even consider clinical or physiological disease related criteria. Future research should concentrate on how to efficiently use resources to achieve the best possible health outcome.

## Abbreviations

AMCP: Academy of Managed Care Pharmacy; HEDIS: Healthcare Effectiveness Data and Information Set; ISPOR: International Society of Pharmacoeconomics and Outcomes Research; JCAHO: Joint Commission on Accreditation of Healthcare Organizations; NBCH: National Business Coalition on Health; NCQA: National Committee for Quality Assurance; NQF: National Quality Forum; OTC: Over the counter; PBM: Pharmacy benefits management; Rx: Prescription drugs; URAC: Utilization Review Accreditation Commission; USA: United States of America; VA: Veterans Affairs.

## Competing interests

Funding: AP. Holtorf, C. McAdam-Marx, G. Oderda are consultants with the Millcreek Outcomes Group, which was a paid consultant to Pfizer in connection with the research and development of the manuscript.

## Authors' contributions

APH and CMAM carried out the data collection and analysis, interpreted the data with the help of all other authors, and drafted the manuscript. BE and GO were involved in the original conceptualization and design of the study, DS, BE and GO supported APH and CMAM in the conceptualization and revisions of the manuscript. All authors read and approved the final manuscript.

## Pre-publication history

The pre-publication history for this paper can be accessed here:



## Supplementary Material

Additional file 1 **Supplemental Table.** Included Study Summaries Click here for file
