# Supplementary material for: Systematic review on quality control for drug management programs: Is quality reported in the literature?
Source: BMC Health Serv Res. 2009 Feb 25;9:38. doi: 10.1186/1472-6963-9-38 (PMC2653499; doi:10.1186/1472-6963-9-38)
Supplement: Additional file 1 — Supplemental Table. Included Study Summaries [file 1472-6963-9-38-S1.doc]

Table 2: Included Study Summaries

|  |  | **Plan Focus** | | **Patient Focus** | | **Treatment Focus** | | **Clinical Outcomes** | | **Prescriber Focus** | | |  |
| --- | --- | --- | --- | --- | --- | --- | --- | --- | --- | --- | --- | --- | --- |
| **n=78** | **Main Study Focus** | **Plan Cost** | **Rx Util.** | **Out of P.$** | **Pt. SF** | **Adhe-rence** | **Lk.Pt. Recei-ving Tx** | **Clin./ Phys. Outc.** | **Med. Res. Util.** | **Rx x-ing.** | **Guideline adh.** | **Workload Prov.** | **No. of end-pnts** |
| Abourjaily 2005(s) | Formulary Restriction, Therapeutic Interchange | √ |  |  |  |  |  |  |  |  |  | √ | 2 |
| Abugosh 2004 | Formulary Restriction |  |  |  |  | √ |  |  |  |  |  |  | 1 |
| Adams 2001 | Formulary Restriction | √ | √ |  |  | √ |  |  |  |  |  |  | 3 |
| Adams 2002 | Formulary Restriction | √ | √ |  |  |  |  |  |  |  |  |  | 2 |
| Artz 2002 | Cost Sharg. |  | √ | √ |  |  |  |  |  |  |  |  | 2 |
| Berger 2007 | Cost Sharg. | √ | √ | √ |  | √ |  |  |  |  |  |  | 4 |
| Briesacher 2004 | Cost Sharg. |  |  |  |  |  | √ |  |  |  |  |  | 1 |
| Brixner 2007 | Cost Sharg. | √ |  | √ |  | √ |  |  |  |  |  |  | 3 |
| Bukstein 2006 | Formulary Restriction, Prior Auth. |  |  |  |  |  |  |  |  |  |  | √ | 1 |
| Carroll 2005 | Multiple Drug Mgmt Programs |  |  | √ |  |  |  |  |  |  |  |  | 1 |
| Christian-Herman 2004 | Formulary Restriction | √ |  | √ |  | √ |  |  | √ |  |  |  | 4 |
| Cox 2001 | Formulary Restriction |  |  |  |  | √ | √ |  |  |  |  |  | 1 |
| Cox 2004 | Formulary Restriction |  |  |  |  | √ | √ |  |  |  |  |  | 3 |
| Desselle 2001 | Formulary Restriction |  |  |  | √ |  |  |  |  |  |  |  | 1 |
| Dunn 2006 | Formulary Restriction; Step Edits |  | √ |  |  |  |  |  |  |  |  |  | 1 |
| Fairman 2003 | Cost Sharg. | √ |  | √ |  | √ |  |  | √ |  |  |  | 4 |
| Feldman 1999 | Prior Auth. | √ |  |  |  |  |  |  |  |  |  |  | 1 |
| Fink 2004 | Descriptive | √ |  |  |  |  |  |  |  |  |  |  | 1 |
| Fischer 2004, | Prior Auth. | √ | √ |  |  |  |  |  |  |  |  |  | 2 |
| Fisher 2003 | Generic Programs | √ |  |  |  |  |  |  |  |  |  |  | 1 |
| Gershovich 2006 | Therapeutic Interchange |  |  |  | √ |  |  |  |  |  |  |  | 1 |
| Gibson 2005 (L) | Formulary Restriction; Cost Sharg. | √ |  |  |  |  |  |  |  |  |  |  | 1 |
| Gibson 2006 | Cost Sharg. |  |  |  |  | √ |  |  |  |  |  |  | 1 |
| Gibson 2006-2 | Cost Sharg. | √ |  | √ |  | √ |  | √ | √ |  |  |  | 5 |
| Gilman 2007 | Cost Sharg. | √ | √ | √ |  |  |  |  |  |  |  |  | 3 |
| Goldman 2006 | Cost Sharg. |  |  |  |  | √ |  |  |  |  |  |  | 1 |
| Harris 2004 | Cost Sharg.; Generic Programs | √ |  |  |  |  |  |  |  |  |  |  | 1 |
| Hartung 2004 | Prior Auth. |  | √ |  |  |  |  |  |  |  |  |  | 1 |
| Hartung 2006 | Preferred Drug List | √ | √ |  |  |  |  |  |  |  | √ |  | 3 |
| Hillman 1999 | Cost Sharg. | √ |  |  |  |  |  |  |  |  |  |  | 1 |
| Hsu 2006 | Formulary Restriction |  |  |  |  | √ | √ | √ | √ |  |  |  | 4 |
| Huskamp 2003 | Cost Sharg. | √ |  | √ |  | √ | √ |  |  |  |  |  | 4 |
| Huskamp 2005 | Cost Sharg. | √ |  | √ |  |  | √ |  |  |  |  |  | 3 |
| Johnson 1997 | Cost Sharg. | √ | √ | √ |  |  |  |  |  |  |  |  | 3 |
| Johnsrud 2007 | Cost Sharg. | √ | √ |  |  |  |  |  |  |  |  |  | 2 |
| Joyce 2002 | Cost Sharg. | √ |  | √ |  |  |  |  |  |  |  |  | 2 |
| Kamal-Bahl 2004 | Cost Sharg. | √ |  | √ |  |  | √ |  |  |  |  |  | 3 |
| Kessler 2007 | Cost Sharg. | √ |  | √ |  | √ |  |  |  |  |  |  | 3 |
| Landon 2007 | Formulary Restriction; Cost Sharg. | √ | √ | √ |  |  |  |  |  |  |  |  | 3 |
| Landsman 2005 | Cost Sharg. |  |  |  |  | √ |  |  |  |  |  |  | 1 |
| Lurk 2004 | Formulary Restriction; Cost Sharg. | √ | √ | √ |  |  |  |  |  |  |  |  | 3 |
| Mager 2007 | Cost Sharg. | √ | √ |  |  |  |  |  |  |  |  |  | 2 |
| Mahoney 2006 | Cost Sharg. |  |  | √ |  |  |  |  | √ |  |  |  | 2 |
| McCombs 2002 | Prior Auth. | √ |  |  |  | √ | √ |  |  |  |  |  | 3 |
| Meissner 2004 | Cost Sharg. | √ | √ | √ |  |  |  |  |  |  |  |  | 3 |
| Meissner 2006 | Therapeutic Interchange | √ |  |  |  |  |  |  |  |  |  |  | 1 |
| Monane 1998 | Cost Sharg. |  | √ |  |  |  |  |  |  |  | √ |  | 2 |
| Motheral 1999 | Pharmacist Cogn. Services | √ | √ | √ |  | √ | √ |  |  |  |  |  | 5 |
| Motheral 2001 | Cost Sharg. | √ |  | √ |  | √ |  |  | √ |  |  |  | 4 |
| Motheral 2004 | Cost Sharg. | √ |  |  | √ |  |  |  |  |  | √ |  | 3 |
| Motheral 2004 | Formulary Restriction |  |  | √ |  | √ | √ |  |  |  |  |  | 4 |
| Mojtabai 2003 | Formulary Restriction |  |  |  |  | √ | √ | √ |  |  |  |  | 3 |
| Murawski 2005 | Preferred Drug List | √ |  |  |  |  |  |  | √ |  |  |  | 2 |
| Nair 2002 | Cost Sharg. |  |  |  | √ |  |  |  |  |  |  |  | 1 |
| Nair 2003 | Cost Sharg. |  |  |  |  | √ |  |  |  |  |  |  | 1 |
| Oh 1999 | Therapeutic Interchange | √ |  |  |  |  |  | √ |  |  |  |  | 2 |
| Olson 2005 | Formulary Restriction |  |  |  | √ |  |  |  |  |  |  |  | 1 |
| Panzer 2005(LM) | Step Edit | √ |  |  |  |  |  |  |  |  |  |  | 1 |
| Rector 2003 | Cost Sharg. |  | √ |  |  |  |  |  |  |  |  |  | 1 |
| Richards 2004(L) | Formulary Restriction; Prior Auth. | √ | √ |  |  |  |  |  |  |  |  |  | 2 |
| Richerson 2001 | Formulary Restriction | √ |  |  |  |  |  | √ |  |  |  |  | 2 |
| Sansgiry 2004 | Preferred Drug List |  |  |  | √ |  |  |  |  |  |  |  | 1 |
| Spence 2006 | Cost Sharg.; Generic Programs |  |  | √ |  |  |  |  |  |  |  |  | 1 |
| Sprague 1998 | Therapeutic Interchange | √ |  |  |  |  |  | √ |  |  |  |  | 2 |
| Sullivan 2005 | Multiple Drug Mgmt Programs | √ |  |  |  |  |  |  |  |  |  |  | 1 |
| Sweet 2001 | Therapeutic Interchange | √ |  |  |  |  |  |  |  |  |  |  | 1 |
| Taira 2006 | Cost Sharg. |  |  | √ |  | √ |  |  |  |  |  |  | 2 |
| Thiebaud 2005 | Multiple Drug Mgmt Programs |  | √ | √ |  |  |  |  |  | √ |  |  | 3 |
| Thomas 2002 | Formulary Restriction; Cost Sharg. | √ |  | √ |  |  |  |  |  |  |  |  | 2 |
| Trygstad 2006 | OTC Coverage | √ |  |  |  |  |  |  |  | √ |  |  | 2 |
| Tseng 2003 | Multiple Drug Mgmt Programs |  |  | √ |  |  |  |  |  |  |  |  | 1 |
| Tseng 2004 | Formulary Restriction; Quantity Level Limit |  | √ | √ | √ | √ | √ |  |  | √ |  |  | 6 |
| Tucker 2001 | Multiple Drug Mgmt Programs | √ |  |  |  |  |  |  |  |  |  |  | 1 |
| Wilson 2005 | Formulary Restriction |  |  |  |  |  | √ |  |  | √ |  |  | 2 |
| Yokohama 2007 | Step Edit | √ | √ |  |  |  | √ |  |  |  |  |  | 3 |
| Zeber 2007 | Cost Sharg. |  | √ | √ |  |  |  |  | √ |  |  |  | 3 |

Abbreviations: Rx Ut.: Drug Utilization, Out of P.$: Out of Pocket Cost, Pt. SF: Patient Satisfaction, Adherence: Adherence, Compliance, Continuation of therapy, Lk. Pt. Receiving Tx: Likelihood of patients receiving treatment, Clin./Phys. Outc.: Clinical or physiological Outcome, Med.Res.Util.: Medical Resource Utilization, Rx x-ing: Drug switching, Guideline adh.: Guideline adherence, Work load Prov.: Workload for Provider, No. of endpnts: Number of endpoints measured, Cost Sharg.: Cost sharing, Prior Auth.: Prior Authorization, Drug Mgmt: Drug management
